# Neural Correlates of Emotional Personality: A Structural and Functional Magnetic Resonance Imaging Study

**DOI:** 10.1371/journal.pone.0077196

**Published:** 2013-11-27

**Authors:** Stefan Koelsch, Stavros Skouras, Sebastian Jentschke

**Affiliations:** Department of Psychology & Cluster Languages of Emotion, Freie Universität, Berlin, Germany; University of Maryland, College Park, United States of America

## Abstract

Studies addressing brain correlates of emotional personality have remained sparse, despite the involvement of emotional personality in health and well-being. This study investigates structural and functional brain correlates of psychological and physiological measures related to emotional personality. Psychological measures included neuroticism, extraversion, and agreeableness scores, as assessed using a standard personality questionnaire. As a physiological measure we used a cardiac amplitude signature, the so-called *E*_κ_ value (computed from the electrocardiogram) which has previously been related to tender emotionality. Questionnaire scores and *E*_κ_ values were related to both functional (*eigenvector centrality mapping*, ECM) and structural (*voxel-based morphometry*, VBM) neuroimaging data. Functional magnetic resonance imaging (fMRI) data were obtained from 22 individuals (12 females) while listening to music (joy, fear, or neutral music). ECM results showed that agreeableness scores correlated with centrality values in the dorsolateral prefrontal cortex, the anterior cingulate cortex, and the ventral striatum (nucleus accumbens). Individuals with higher *E*_κ_ values (indexing higher tender emotionality) showed higher centrality values in the subiculum of the right hippocampal formation. Structural MRI data from an independent sample of 59 individuals (34 females) showed that neuroticism scores correlated with volume of the left amygdaloid complex. In addition, individuals with higher *E*_κ_ showed larger gray matter volume in the same portion of the subiculum in which individuals with higher *E*_κ_ showed higher centrality values. Our results highlight a role of the amygdala in neuroticism. Moreover, they indicate that a cardiac signature related to emotionality (*E*_κ_) correlates with both function (increased network centrality) and structure (grey matter volume) of the subiculum of the hippocampal formation, suggesting a role of the hippocampal formation for emotional personality. Results are the first to show personality-related differences using eigenvector centrality mapping, and the first to show structural brain differences for a physiological measure associated with personality.

## Introduction

Personality is a construct with substantial emotional components. For example, neuroticism relates to tendencies for the experience of negative emotions such as depression and anxiety, extraversion relates to behaviour of social approach and avoidance, and agreeableness relates to tender-mindedness on the one side, and to emotional coldness on the other. Correspondingly, differences in personality (such as differences in neuroticism, extraversion and agreeableness) have been reported to be related to functional differences in brain structures implicated in emotion, e.g. orbitofrontal cortex (OFC), amygdala, cingulate cortex, insula, and hippocampal formation: Functional neuroimaging studies have suggested associations between neuroticism and neural activity in anterior cingulate cortex (ACC) [1]–[4], insula [5], [6], anterior fronto-median cortex [4], [7], and amygdala (during fear learning) [8]. Extraversion has been reported to be related to neural activity in the striatum [9]–[11], ACC [12]–[14], OFC [5], [6], and amygdala (in response to positive stimuli) [10], [15], [16]. Consistent findings for other personality dimensions (agreeableness, openness, and conscientiousness) are rather sparse, except that agreeableness is perhaps linked to activity in the right lateral PFC (taken to be related to emotion regulation) [17], and that openness has been linked to executive functions of the prefrontal cortex [18] as well as to serotonergic activity within the striatum [19] (for an overview see Table 1A).

**Table 1:** Overview of functional (A) and structural (B) brain differences reported in studies using subjective measures of personality factors.

Study	n	OFC	amyg	CC	hipp	insula	striatum	aFMC	PFC	cer
***A: functional studies***										
Eisenberger et al. [1] 1	17			N					E, N	
Tauscher et al. [2]	19			N						
Frokjaer et al. [3]	83			N						
Brück et al. [4]	24		N	N				N		
Paulus et al. [5]	17					N				
Deckersbach et al. [6]	20	E				N				
Britton et al. [7]	12							N		
Hooker et al. [8] 2	12		E, N		N					
Fischer et al. [9] 2	30						E			
Canli et al. [10]	14		E	E			E		N	
Cohen et al. [11]	17	E					E			
Kumari et al. [12] 3	11			E					E	
Canli et al. [13]	12			E						
Haas et al. [14]	26			E						
Canli et al. [15] 2	15		E							
Vaidya et al. [16]	12		E							
Haas et al. [17]	48								A	
Kalbitzer et al. [19]	50						O			
Chan et al. [120] 9	25		N							N
Cunningham et al. [121] 9	21		N							
Brühl et al. [122] 2	16								E	
Simon et al. [123] 10	24		E	E			E			
***B: structural studies***										
DeYoung et al. [20]	116	E		N, A	N		N	N	N, A, C	
Cremers et al. [21]	65	E	E							
Rauch et al. [22] 4	14	E								
Omura et al. [23]	41		E, N							
Wright et al. [24]	29								E	
Forsman et al. [25] 5	34						E		E	
Gardini et al. [26] 6	85	N, E					E			
Wright et al. [27]	28	N						N	E	
Iidaka et al. [28] 6	56		N				E			
Barros-Loscertales et al. [29] 7	63		N		N					
Yamasue et al. [30] 6	183				N				N	
Schutter et al. [31] 8	38									N
Hu et al. [32]	62	O, A		O						A

The studies listed in the outermost left column used five factor personality inventories (neuroticism, extraversion, openness, agreeableness, conscientiousness), or similar constructs (see table footnotes). The second left column indicates the sample size. Only brain structures reported by at least two studies are listed. Note the relatively low replicability of findings. Abbreviations: OFC: orbitofrontal cortex, amyg: amygdala; CC: cingulate cortex; hipp: hippocampal formation; aFMC: anterior fronto-median cortex; PFC: prefrontal cortex; cer: cerebellum.

1 Only neuroticism, extraversion, and self-consciousness were assessed.

2 Only neuroticism and extraversion were assessed.

3 Only neuroticism, extraversion, and psychoticism were assessed.

4 Only neuroticism and extraversion were evaluated.

5 A five-factor model of Cattel's 16 Personality Factor questionnaire was used.

6 Cloniger's Three-dimensional Personality Questionnaire was used, including harm avoidance (which is conceptually related to neuroticism) and reward-dependence (which is conceptually related to extraversion).

7 Only the Sensitivity to Punishment scale (which is conceptually related to neuroticism) of the Behavioral Inhibition System was used.

8 Only neuroticism-subscales (anxiety, depression) were assessed.

9 Only neuroticism was assessed.

10 Behavioral Inhibition/Behavioral Approach Activation Scales were used (which is conceptually related to extraversion).

Correspondingly, anatomical differences in these brain structures were reported to be associated with personality (see also Table 1B): Extraversion has been associated with volume [20], [21] and cortical thickness of the OFC [22]; other studies have associated extraversion with volume of the right amygdala [21] or gray matter concentration of the left amygdala [23], and with cortical thickness of the right dorsolateral prefrontal cortex (DLPFC) [24]. Moreover, extraversion and reward-dependence (which is conceptually related to extraversion) have been reported to be negatively correlated with volume of the caudate nucleus [25], [26]. Neuroticism and harm avoidance (which is conceptually related to neuroticism) have also been associated with volume [26] and cortical thickness [27] of the OFC. In addition, neuroticism has been associated with volume of the left amygdala (in females) [28] and the right amygdala [23]. Similarly, a study by Barros-Loscertales et al. [29] reported increased right amygdalar volume (as well as increased left entorhinal volume) in individuals with increased sensitivity to punishment (as measured with the Behavioural Inhibition System). Furthermore, a study by DeYoung et al. [20] found an association between neuroticism and mid-cingulate volume, a study by Yamasue et al. [30] reported that grey matter volume in the (right) hippocampal formation was negatively related to harm avoidance, and Schutter et al. [31] observed that neuroticism correlated negatively with cerebellar volume. Only few studies have reported relations between brain structure and agreeableness, openness and conscientiousness. Agreeableness was observed to be related to DLPFC volume (taken to be related to the processing of intentions and mental states of others) [20] and cerebellar volume [32]. Openness has been linked to orbitofrontal and (dorsal) ACC volume [32], and conscientiousness has also been linked to DLPFC volume (taken to be related to processes of executive control) [20].

The mentioned studies reporting relations between personality and both activity and morphology of limbic/paralimbic brain structures appear to support the notion of a tight association between personality and emotionality. Note, however, that the replicability of the findings of the mentioned studies is relatively low (for an illustration see Table 1). One reason for such relatively low replicability might be that the mentioned studies assessed personality using subjective measures (questionnaires). Such psychological measures have the advantage of a direct semantic relation between the questionnaire items and the construct of a personality factor. The subjectivity of these measures, however, also bears the problem of potential biases such as socially desirable responding, inaccuracies in self-perception, self-favouring tendencies, self-deception, and moralistic bias [33]–[37]. Moreover, it cannot be guaranteed that the inner states described by emotion-words in questionnaires (such as “very happy”) refer to the same subjective experience in different individuals, or even in the same individual at different points in time (see also Wittgenstein's arguments against the idea of a “private language”) [38].

### Physiological measures of personality

In addition to subjective measures of personality (obtained with a standard personality questionnaire), the present study also used a physiological measure taken to reflect emotional personality. A substantial amount of research associating personality with biological parameters such as levels of neurotransmitters, hormones, and autonomic activity [39]–[45] opens the interesting possibility of using identified biomarkers as indices of personality. Such physiological markers have the advantage that they are not dependent on subjective ratings. However, a disadvantage is that physiological measures can be biased by factors unrelated to personality (such as general health status, inflammation, current mood, or circadian rhythm). Moreover, they have the disadvantage that there is no direct semantic link between a physiological measure and a personality construct. Hence, it is arguable to which degree a physiological personality measure is actually indicative of a specific personality trait.

**Figure 1:**
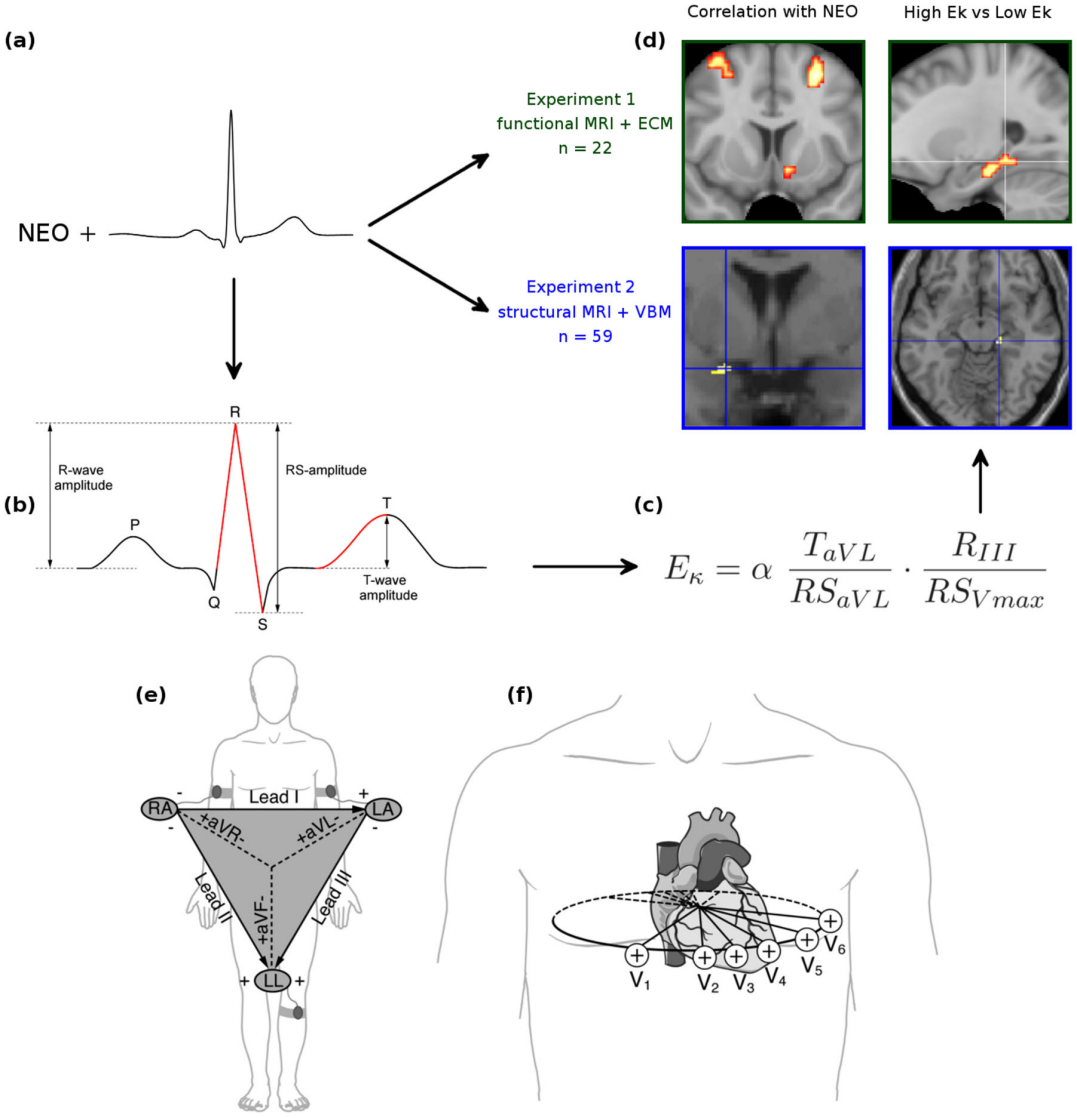
Modulatory influence of psychological factors on reginal cardiac activity. (**a**) From each of *n* = 81 participants (22 in Experiment 1, and 59 in Experiment 2), NEO scores as well as a 12-lead rest electrocardiogram (ECG) were obtained. (**b**) From each ECG, absolute values of mean R-, RS-, and T-wave amplitudes were measured electronically (separately for the leads aVL, RIII, and all chest leads), and then computed according to the equation shown in (**c**), resulting in a single *E*_κ_ value for each participant (*E*_κ_ is taken to reflect tender emotionality). *E*_κ_ values were computed using the absolute amplitude values of the T-wave of aVL (*T_aVL_*), the RS-wave of aVL (*RS_aVL_*), the R-wave of III (*R_III_*) and the maximal RS-wave measured at any of the chest leads (*RS_Vmax_*). For better readability, *E*_κ_ values were scaled with a factor of α = 10. (**d**) Functional magnetic resonance (MR) images were obtained from the participants of Experiment 1, and structural MR images were acquired from the participants of Experiment 2. In Experiment 1, Eigenvector Centrality Maps (ECMs) were computed for each participant. ECMs were then correlated with NEO scores of participants (upper left panel of **d**), and compared between groups of individuals with higher and lower *E*_κ_ values (upper right panel of **d**). Likewise, structural data obtained in Experiment 2 were correlated with NEO scores (lower left panel of **d**), and compared between groups of individuals with higher and lower *E*_κ_ values (lower right panel of **d**). The bottom panel illustrates standard ECG leads: The six extremity leads (I, II, III, aVL, aVR, aVF) record voltage differences by means of electrodes placed on the limbs (**e**). The triangle shows the spatial relationships of the extremity leads, which record electrical voltages onto the frontal plane of the body. The six chest leads (V1–V6) record voltage differences by means of electrodes placed on the chest wall (**f**). The oval indicates spatial relationships of the six chest leads, which record electrical voltages transmitted onto the horizontal plane.

As a physiological index of emotional personality, we used a cardiac amplitude signature. Amplitudes of the electrocardiogram (ECG, see also Figure **1**) reflect regional cardiac activity, which is modulated by a number of psychological factors (summarized in Box 1). Therefore, it has been proposed that cardiac amplitude signatures can be used as biomarker for personality [46]. The cardiac amplitude signature used in the present study is characterized by the relation of four amplitude values of the ECG. The computation of these amplitude values according to the equation shown in Figure **1**c results in a value for each individual, referred to as *E*_κ_ value. This *E*_κ_ measure has previously [46] been obtained using discriminant function analysis on ECG amplitude data to differentiate groups of individuals with higher and lower scores of *tender emotionality* (as indicated by interviews and self-reports). The construct of tender emotionality refers to the tendency to experience positive emotions (including feelings of joy and happiness), attachment-related emotions (such as love), compassion and empathy. Persistent lack of tender emotion (also referred to as emotional coldness, or flattened affect) is a clinically relevant symptom in schizoid personality disorder and schizophrenia. In that study [46] data from two functional neuroimaging experiments showed stronger BOLD responses to emotional stimuli in amygdala and hippocampal formation in individuals with high *E*_κ_ (compared to individuals with low *E*_κ_).

**Box 1:**
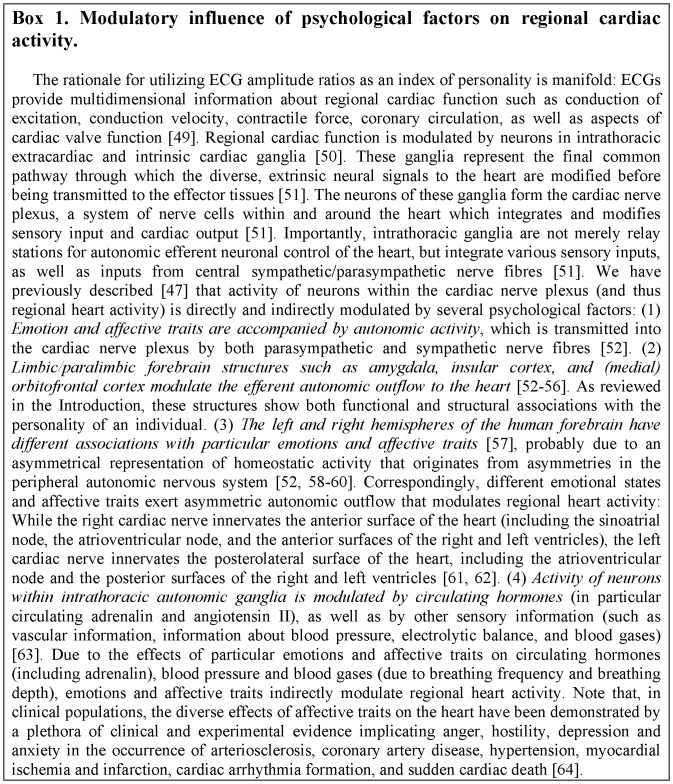
Illustration of procedure and data analysis.

The notion that *E*_κ_ reflects aspects of the emotional personality of an individual was supported by a subsequent study [47] in which individuals with higher *E*_κ_ (assumed to reflect higher tender emotionality) had lower neuroticism scores, higher scores of positive emotion, and tended to have higher extraversion scores (personality scores were measured using the NEO-FFI and NEO-PI-R inventories [48]). Note that, because there is no direct semantic link between tender emotionality and *E*_κ_, it would be problematic to simply equate the two (as outlined above). Nevertheless, in the present study we tested whether *E*_κ_ as a physiological index of emotional personality would show relations to brain function and brain structure.

### The present study

In the present study, we used both psychological and physiological measures related to emotional personality as independent variables to investigate neural correlates of emotional personality in both functional and structural magnetic resonance imaging (MRI) data (Figure **1**). Psychological measures included the *neuroticism*, *extraversion* and *agreeableness* scales of the Neuroticism-Extraversion-Openness Five Factor Inventory (NEO-FFI) [48], the extraversion-facets *positive emotion* and *warmth*, as well as the agreeableness-facet *tender-mindedness* (as obtained with the revised version of the NEO Personality Inventory; NEO-PI-R) [33]. As physiological measure we used the (cardiac) *E*_κ_ index. In Experiment 1, subjects performed a functional neuroimaging session, and functional data were then correlated with both psychological and physiological personality measures. In Experiment 2, structural brain scans were obtained from an independent sample of subjects, and brain-volumetric data were then correlated with both psychological and physiological personality measures. This enabled us (a) to compare consistency between functional and structural findings, (b) to determine the replicability of previous results with regard to psychological and physiological measures, (c) to relate the physiological data to the psychological data, and (d) to compare neurological results obtained with psychological or physiological data as independent variables.

## Experiment 1: Functional MRI Using Eigenvector Centrality Mapping (ECM)

In Experiment 1, fMRI data were analysed using *Eigenvector Centrality Mapping* (ECM) [65] to identify voxels that show high eigenvector centrality within neuro-functional small-world networks [66]. ECM attributes a centrality value to each voxel in the brain such that a voxel receives a large value if it is strongly correlated with many other nodes that are themselves central within the network (Google's Page-Rank algorithm is a variant of eigenvector centrality). ECM thus indicates “computational hubs” of neural small-world networks [67]. For example, a previous study using a within-subjects design compared data of resting state scans of subjects when they were in states of hunger or satiety [65] (that study reported that centrality values were higher during the hungry state in the posterior cingulate cortex and the precuneus). ECM is reminiscent of resting-state fMRI [68], with the important difference that ECM can be computed on any functional neuroimaging data (i.e., not necessarily resting state data).

With regard to correlations between NEO scores and functional neuroimaging data, we hypothesized (based on the most consistent findings, as reviewed above) that extraversion scores would correlate with neural activity in the striatum [9]–[11] and the ACC [12]–[14]. Moreover, we tested whether neuroticism would also correlate with activity in the ACC [1]–[4]. Based on our previous study [46], in which individuals with low *E*_κ_ (taken to reflect low tender emotionality) showed reduced neural activity in amygdala and hippocampal formation (in response to emotion-evoking music stimuli), we aimed to test in the present study whether individuals with lower *E*_κ_ would show lower centrality values in hippocampal formation and amygdala. With regard to associations between NEO scores and *E*_κ_ values, we expected that individuals with higher *E*_κ_ values (reflecting higher tender emotionality) would have lower neuroticism scores, higher extraversion scores, and higher scores of positive emotion than individuals with lower *E*_κ_ values (as in our previous study [47]). Because warmth and tender-mindedness are conceptually related to the construct of tender emotion, we hypothesized that scores of these facets, as well as of agreeableness, would be higher in individuals with higher *E*_κ_ values than in individuals with lower *E*_κ_ values.

### Materials and methods

#### Ethics statement

All subjects gave written informed consent. The study was conducted according to the Declaration of Helsinki and approved by the ethics committee of the School of Life Sciences and the Psychology Department of the University of Sussex.

#### Participants

Data were obtained from 22 individuals (aged 19–31 years, *M* = 23.50, *SD* = 3.36, 12 females). Exclusion criteria were left-handedness, consumption of alcohol or caffeine exceeding one litre during the 24 hours prior to testing, poor sleep during the previous night, past diagnosis of a neurological, audiological or psychiatric disorder (according to self-report), and abnormal brain anatomy (as diagnosed by a radiologist).

#### Experimental procedure

*Psychological (NEO) measures.* From each participant, we obtained scores of the personality scales *Neuroticism*, *Extraversion*, and *Agreeableness* (each with 12 Items) from the German translation of the NEO Five Factor Inventory (NEO-FFI) [48], [68]. Moreover, scores of the personality facets *warmth*, *positive emotion* and *tender-mindedness* (each with 8 Items) from the German translation of the revised version of the NEO Personality Inventory (NEO-PI-R) [48], [69] were obtained. *Warmth* and *positive emotion* are facets of the extraversion scale, and *tender-mindedness* is a facet of the agreeableness scale. These facets were used because they are more closely related to the construct of tender emotionality than other NEO-PI-R facets: *positive emotion* is related to a tendency to experience positive emotions, *tender-mindedness* is related to an attitude of sympathy for others, and *warmth* is related to interest in and friendliness towards others [48]. The number of items amounted to 54 Items (note that because the NEO-FFI is a short version of the NEO-PI-R, twelve items of the used NEO-FFI scales are identical to those of the used NEO-PI-R facets).

*Physiological (cardiac) measures.* A 12-leads resting ECG (2–3 min duration) was acquired from each participant in supine position under standard conditions [70]. ECGs were recorded (with disposable electrodes) with high resolution (sampling rate: 1000 Hz, resolution: 22 bit, without on-line filtering) using a cardio 100 system (custo med GmbH, Ottobrunn, Germany). ECGs and NEO data were obtained directly before the functional neuroimaging experiment.

*Functional neuroimaging experiment.* For the functional neuroimaging session we employed an experiment on music and emotion using joy, fear and neutral musical stimuli (joy and fear stimuli are listed in Table S1) [71]. We chose an emotion experiment for data acquisition to specify in which brain structures personality-related functional differences were dependent on the emotional state of subjects. Joy and fear were chosen because they represent basic emotion categories motivating approach and avoidance behaviours.

Each experimental stimulus had a duration of 30 s, and there were 8 stimuli of each emotion category (joy, fear, neutral; for details see Supporting Methods). Prior to the fMRI session, participants were presented with short (12 s) versions of each stimulus to obtain familiarity ratings: Participants rated their familiarity with each piece on a four-point scale (ranging from “To my knowledge I have never heard this piece before”, to “I know this piece, and I know who composed, or performed it”). Participants were then trained on the emotion-rating procedure using 12 s long excerpts of musical pieces that did not belong to the stimulus set used in the fMRI scanning session: At the end of each excerpt, participants indicated how they felt with regard to valence (pleasant/unpleasant), arousal (calm/excited), joy and fear. That is, participants provided ratings about how they felt, and not about which emotion they thought each song was supposed to express [72]. Ratings were obtained with 6-point Likert scales (ranging from “not at all” to “very much”), and the time interval for each rating was 3 s (amounting to a duration of the entire rating procedure of 12 s).

In the fMRI experiment, stimuli were pseudorandomly intermixed so that no more than two stimuli of each stimulus category (joyful, fearful, neutral) followed each other. Moreover, the entire stimulus set was presented twice (i.e., 16 stimuli per category were presented). Participants were asked to listen to the musical stimuli with their eyes closed. Each musical stimulus was followed by an interval of 2 s in which a beep tone signalled participants to open their eyes and to commence the emotion-rating procedure (as described above). Each rating period was followed by a 4 s rest period, amounting to a total length of 48 s per trial, and a duration of the functional neuroimaging experiment of about 39 min.

Musical stimuli were presented using Presentation (version 13.0, Neurobehavioral systems, Albany, CA, USA) via MRI compatible headphones (under which participants wore earplugs). Instructions and rating screens were delivered through MRI compatible liquid crystal display goggles with an integrated eyetracker (Resonance Technology Inc., Northridge, CA, USA). The eyetracker was used by the experimenters to control that participants followed the instructions regarding opening and closing their eyes.

#### MR scanning

Scanning was performed with a 3T Siemens Magnetom Trio scanner (Siemens Medical Systems, Erlangen, Germany). Continuous Echo Planar Imaging (EPI) was used with a TE of 30 ms and a TR of 2,000 ms. Slice-acquisition was interleaved within the TR interval. The matrix acquired was 64×64 voxels with a Field Of View (FOV) of 192 mm, resulting in an in-plane resolution of 3 mm. Slice thickness was 3 mm with an interslice gap of 0.6 mm (37 slices, whole brain coverage). The acquisition window was tilted at an angle of 30 degrees relative to the AC-PC line in order to minimize susceptibility artifacts in the orbitofrontal cortex [73]–[75].

#### ECG data analysis

ECG-wave detection and measurement of ECG amplitude values was performed electronically using the in-house software package Kardionoon [46] (publicly available at http://sourceforge.net/projects/kardionoon) and visually inspected by the first author. The amplitude measurements are described in detail elsewhere [46]; in short, for each lead of each participant, all P-, R-, S-, and T-wave peaks were identified in the raw ECG, and artifact-free waves were then averaged separately for each lead to obtain ECG waves representative for each participant (i.e., for a 2-min artifact-free ECG of a subject with a heart rate of 60 beats per minute, 120 P-, 120 R-, 120 RS-, and 120 T-waves were averaged, separately for each wave, and for each of the twelve ECG leads). From these averaged ECG waves, absolute amplitude values of P-, R-, RS-, and T-waves were measured electronically; R-wave amplitudes were measured with respect to the baseline of the averaged ECG cycle (see also Figure 1b for illustration), T-wave amplitudes were measured with respect to the first plateau-like wave shape preceding the T-wave peak [46]. Moreover, to reduce potential bias introduced by slightly different placement of chest electrodes, the maximal value of each of the four ECG waves (P-, R-, RS-, and T-wave) measured at any of the chest leads were also computed. Then, the *E*_κ_ value was computed for each subject according to the equation shown in Figure **1**c. All ECG analyses were carried out blinded, that is, without knowledge about the NEO scores nor (f)MR images of any participant.

#### Functional MRI data analysis

Functional MRI data were processed using LIPSIA 2.1 [76]. Data were corrected for slicetime acquisition and normalized into MNI-space-registered images with isotropic voxels of 3 cubic millimetres. A temporal highpass filter with a cutoff frequency of 1/90 Hz was applied to remove low frequency drifts in the fMRI time series, and a spatial smoothing was performed using a 3D Gaussian kernel and a filter size of 6 mm FWHM.

Eigenvector Centrality Maps (ECMs) were first computed on each participant's entire fMRI dataset, treating the entire functional session as one time period of interest (that is, the entire experiment was calculated as one single trial for the ECM analysis, and only one ECM image was calculated for each participant). Neuroticism, extraversion, and agreeableness scores were then used as regressors of interest (with age and gender as covariates of no interest) in a second level design matrix that served for regressions with the NEO variables. Regressions were used because NEO scores did not deviate from a standard normal distribution (as indicated by Shapiro-Wilk-Tests, see Table S2). By contrast, *E*_κ_ values significantly deviated from a normal distribution (*p* = 0.02 according to the Shapiro-Wilk Test of Normality). Therefore, *E*_κ_ values were dichotomized using a median-split, and eigenvector centrality maps were compared between the resulting two groups (11 individuals with higher, and 11 individuals with lower *E*_κ_). Age and gender were used as covariates of no interest in the second level design matrix for a voxel-wise two-samples *t*–test. All second-level results were corrected for multiple comparisons by the use of cluster-size and cluster-value thresholds obtained by Monte Carlo simulations with a significance level of *p*<0.05 [77] (voxel-wise threshold before applying the Monte Carlo simulation was *z* = 2.326).

To specify whether (a) correlations between brain activations and personality scores and (b) differences in brain activation between *E*_κ_ groups were independent of the emotional state of subjects, we also computed ECMs separately for each music epoch (and for each participant). Thus, 16 ECMs per emotion category (joy, fear, neutral) were computed per participant. Then, for each emotion category the 16 ECMs were averaged into an average ECM per participant (that is, for each participant one average ECM was computed for joy, another one for fear, and a third one for neutral). Subsequently, the neuroticism, extraversion, and agreeableness scores were used as regressors of interest (with age and gender as covariates of no interest) for the average ECMs of each emotion (joy, fear, neutral) in a second level design matrix. Additional second-level analyses were computed to compare the average ECMs of each emotion condition (joy, fear, neutral) between *E*_κ_ groups. Finally, the maximal *z*-values were extracted for those structures in which the first second-level ECM analysis (which used the entire fMRI session as one single trial per participant) indicated significant personality effects. Thus, group results were first obtained with ECMs calculated for the entire fMRI session, and then these results were further investigated using ECMs calculated separately for each emotion condition (joy, fear, neutral).

#### Comparison of NEO and ECG data

In addition to comparing the functional neuroimaging data between groups with higher and lower *E*_κ_, we also compared NEO scores between these two groups. For all six NEO scores we had directed hypotheses: individuals with higher *E*_κ_ values were expected to show lower neuroticism, higher extraversion, higher agreeableness, as well as higher positive emotion, tender-mindedness and warmth (see also Introduction). Thus, the significance level for Bonferroni-corrected one-sided tests was 0.05/6 * 2 = 0.017.

### Results

#### Relations between cardiac (ECG) and psychological (NEO) data

NEO scores were compared between groups of individuals with high and low *E*_κ_ values (i.e., *E*_κ_ values above and *E*_κ_ values below the median) according to the directed hypotheses stated in the Introduction and Methods. Individuals with higher *E*_κ_ had significantly (Bonferroni-corrected) higher extraversion scores compared to those with lower *E*_κ_ (*t* = 2.5, *p* = .01). Moreover, individuals with higher *E*_κ_ had significantly (Bonferroni-corrected) higher scores of positive emotion (*t* = 2.5, *p* = .01, for details and complete data see Table S3).

#### Relations between behavioral ratings and ECG/NEO data

During the experiment, subjects rated at the end of each music stimulus how they felt with regard to valence, arousal, joy, and fear (see *Methods*). To investigate how these emotion ratings were influenced by the different stimulus categories (joy, fear, neutral), an ANCOVA was computed with the within-subjects factor stimulus category (joy, fear, neutral). This analysis indicated main effects of valence (F(2, 20) = 83.76, p<0.001), arousal (F(2,20) = 20.92; p<0.001), joy (F(2, 20) = 196.88, p<0.001), and fear (F(2,20) = 84.97, p<0.001). These results reflect that the stimulus material evoked distinct emotional effects in our participants.

To investigate whether emotion ratings differed between the *E*_κ_ groups (i.e., between individuals with *E*_κ_ values above and *E*_κ_ values below the median), an ANCOVA was carried out with the within-subjects factor stimulus category (joy, fear, neutral) and *E*_κ_ group as between-subjects factor. Results indicated main effects of *E*_κ_ group for arousal (F(1,20) = 6.34, p = 0.020), and fear (F(1,20) = 5.95, p = 0.024). These main effects originated from arousal and fear ratings being higher in the group of individuals with higher *E*_κ_ values.

Finally, to investigate whether emotion ratings were modulated by the NEO scores, an ANCOVA was computed with factor stimulus category, and NEO scores as covariates (neuroticism, extraversion, agreeableness). Results indicated a main effect of extraversion scores for joy ratings (F(1,18) = 5.40, p = 0.032), resulting from lower joy ratings for the fear and neutral stimuli in participants with high extraversion. A main effect of neuroticism scores was marginally significant (F(1,18) = 3.04, p = 0.098) for fear ratings, resulting from fear ratings being higher in participants with higher neuroticism scores. No main effect of agreeableness was observed. Nevertheless, there were interactions of emotion category with the agreeableness scores for arousal (F(2,17) = 4.26, p = 0.032), valence (F(2,17) = 11.91, p = 0.001), and joy (F(2,17) = 8.55, p = 0.003), reflecting that ratings of participants with higher agreeableness scores were more in accordance with the stimulus categories (e.g., they gave higher joy ratings for joy stimuli, and lower joy ratings for fear stimuli, compared to individuals with low agreeableness scores).

#### Psychological (NEO) measures and ECM data

**Figure 2:**
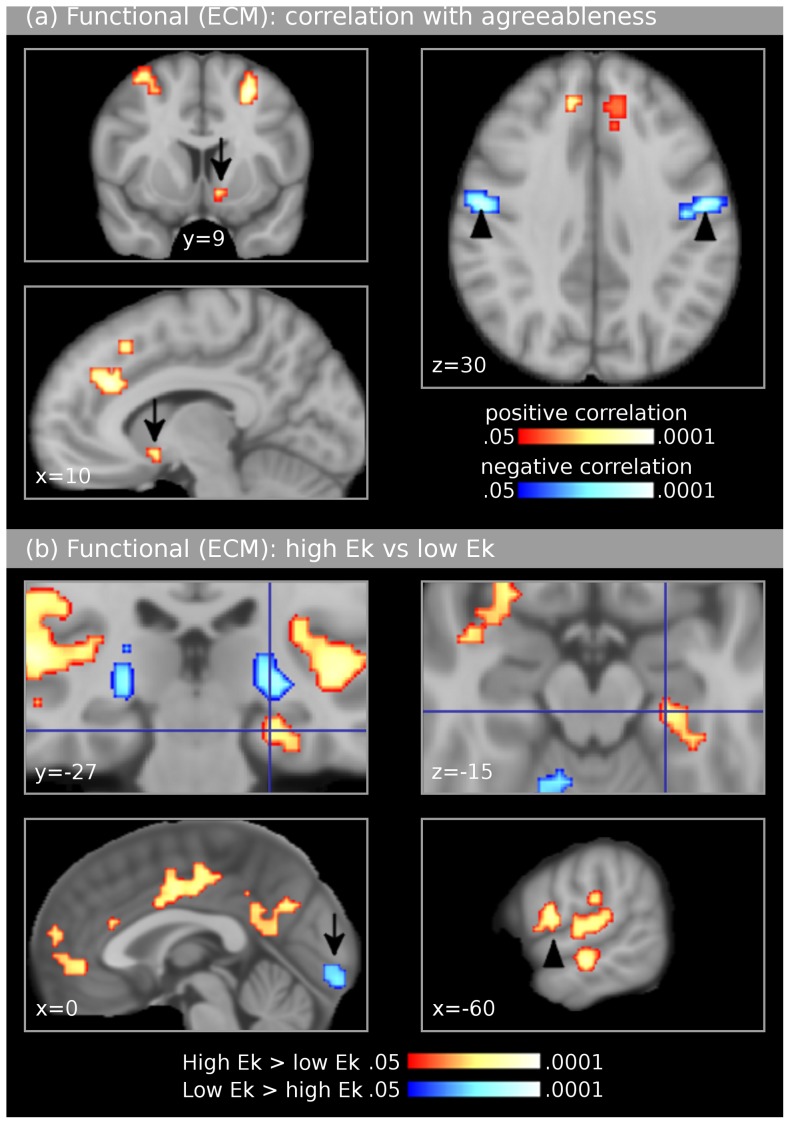
Experiment 1: Functional neuroimaging data (Eigenvector Centrality Mapping). Results were controlled for age and gender, and corrected for multiple comparisons (*p*<.05). Images are shown in neurological convention. The upper panel (**a**) shows results of correlations of Eigenvector Centrality Maps (ECMs) with agreeableness scores. For each participant, one ECM was computed for the entire fMRI session. Positive correlations (shown in red-yellow colours) were found in the posterior superior frontal sulcus bilaterally (upper left image), the ventral striatum/nucleus accumbens (arrows in left upper and lower image), and in the anterior cingulate cortex (lower left image). Negative correlations (shown in blue) were observed in the central sulcus (arrowheads in right image). The bottom panel (**b**) shows the comparison of ECMs between groups of participants with higher and lower *E*_κ_ values. Individuals with higher *E*_κ_ (taken to reflect higher tender emotionality) showed higher centrality values in the subiculum of the right hippocampal formation (crosshairs in upper images), in the auditory cortex bilaterally, in both anterior and posterior cingulate cortex (lower left image), the anterior fronto-median cortex (lower left image), and the Rolandic operculum (arrowhead in lower right image). The group with lower *E*_κ_ showed higher centrality values in the lateral geniculate body bilaterally (blue clusters in upper left image) as well as in the visual cortex (V1, blue cluster in lower left image).

Results of voxel-wise correlations between Eigenvector Centrality Maps (ECMs, computed for the entire fMRI session, see Methods) and scores of each of the NEO scales are listed in Table 2 and shown in Figure **2**a. Significant positive correlations with agreeableness scores were observed in the right ventral striatum/nucleus accumbens, ACC, the rostral cingulate zone extending into the pre-SMA of area 6, and dorsolateral prefrontal cortex bilaterally (posterior superior frontal sulcus and superior frontal gyrus, BA 8). Significant negative correlations were found in the central sulcus (face area of either area 3b or area 4).

**Table 2:** Results of agreeableness regression.

Anatomical structure	MNI coord.	cluster size (mm^3^)	*z*–value: max (mean)	*joy*	*fear*	*neutral*
r post. SFS, area 8	27 9 55	1269	4.03 (2.84)	2.58	2.21	2.57
l post. SFS, area 8	−33 6 67	918	2.95 (2.58)	2.35	2.94	2.33
r SFG, area 8	18 21 61	1674	3.02 (2.54)	3.71	2.76	3.35
l SFG, area 8	−18 27 61	1053	3.26 (2.72)	3.58	2.75	3.67
pre-SMA, area 6	6 18 52	864	3.26 (2.65)	3.72	2.00	2.96
l median wall, area 32	−9 39 40	999	3.06 (2.56)	2.89	3.40	2.32
l ACC, area 29^1^	−9 33 21	–	2.84	2.81	3.46	2.84
r ACC, area 29	12 30 31	1566	3.71 (2.81)	3.80	3.58	2.65
r caudate nucleus	5 6 −5	648	3.17 (2.60)	2.81	2.70	2.81
r ventral striatum (NAc)^2^	11 10 −10	–	2.39	2.12	2.11	2.43
l central sulcus	−57 −9 43	945	−3.37 (−2.71)	−2.88	−2.50	−2.86
r central sulcus	57 −6 31	1350	−3.55 (−2.80)	−3.42	−2.61	−2.24

The table shows results of voxel-wise regressions between Eigenvector Centrality Maps (ECMs, controlled for age and gender) and agreeableness scores, corrected for multiple comparisons (*p*<.05). MNI-coordinates, cluster sizes, and *z*-values refer to the analysis in which one single ECM per participant (calculated for the entire fMRI session) entered the second-level analysis. The three columns on the right indicate maximum *z*-values in the respective structures when ECMs were computed separately for each emotion condition (joy, fear, and neutral, see *Methods*). Abbreviations: l: left; r: right; post.: posterior; ACC: anterior cingulate cortex; MNI: Montreal Neurological Institute; NAc: nucleus accumbens; SFG: superior frontal gyrus; SFS: superior frontal sulcus; SMA: supplementary motor area.

1 The cluster with the peak voxel in area 32 had another local maximum in the ACC.

2 The cluster with the peak voxel in the r caudate had another local maximum in the NAc.

To investigate whether these correlations were dependent on the emotional states of subjects, we also computed average ECMs for each emotion condition separately (see *Methods*). Correlations between these ECMs and agreeableness scores showed that, in all structures listed in Table 2, the significance values of the observed correlations were similar for joy, fear, and neutral (see Table 2 for details; note that all significance values were above *z* = 1.96, corresponding to *p* = .05).

The ECMs computed for the entire fMRI session did not show significant correlations (corrected for multiple comparisons, *p*<.05) with neuroticism, nor with extraversion. However, when testing our directed hypotheses (stated in the Introduction) using uncorrected regression analyses (thresholded at *p* = .001 and a voxel extent of 10 voxels), we found a correlation between neuroticism and centrality values in the cingulate sulcus at the border of the rostral and caudal cingulate zone (MNI coordinate: −2, 7, 43; *z* = 3.4).

#### Physiological (cardiac) measures and ECM data

For the comparison of ECMs between individuals with high and low *E*_κ_, subjects were split into two groups based on a median split of *E*_κ_ values (i.e., *E*_κ_ values above and *E*_κ_ values below the median). Group differences were first investigated using the ECMs computed for the entire fMRI session. The results of this comparison are listed in Table 3 and shown in Figure **2**b (data were controlled for age and gender, and results were corrected for multiple comparisons, *p*<.05). A difference between groups was found in the subiculum of the right hippocampal formation (MNI coordinate: 23, −27, −15, 90% probability for subiculum according to the cytoarchitectonic maps provided by Amunts et al., [78]), in which centrality values were significantly higher in the group with higher *E*_κ_ compared to the group with lower *E*_κ_ (see crosshair in Figure **2**b). Figure **2**b also shows significantly higher centrality values in the group with higher *E*_κ_ in the auditory cortex bilaterally, both anterior and posterior cingulate cortex, the anterior fronto-median cortex, and the Rolandic operculum (for full list see Table 3). The group with lower *E*_κ_ showed higher centrality values in the lateral geniculate body bilaterally as well as in the visual cortex (V1).

**Table 3:** Results of the ECM constrasts high *E*_κ_ vs. low *E*_κ_.

Anatomical structure	MNI coord.	cluster size (mm^3^)	*z*–value: max (mean)	*joy*	*fear*	*neutral*
**High ** ***E*** **_κ_>low ** ***E*** **_κ_**						
r parahipp. g.	28 −34 −15	1755	3.36 (2.73)	2.49	3.09	2.72
r parahipp. g. (SUB, 100%)^1^	25 −25 −16	–	3.23	3.63	2.95	1.59
l ACC	−3 27 25	297	3.64 (2.76)	2.09	2.58	2.99
r ACC	3 42 1	3618	3.06 (2.55)	2.78	2.89	3.13
r aFMC^2^	3 60 5	–	2.75	2.61	2.78	2.96
r post. cingulate g.	12 −42 46	14040	4.12 (2.76)	3.40	4.20	2.27
r insula	45 0 −2	1026	3.23 (2.65)	3.33	3.57	2.54
l IFG, pars orbitalis	−36 27 −20	1863	3.26 (2.59)	1.94	3.80	2.70
l STG (TE 1.0, 30%)	−54 −27 10	14472	3.99 (2.82)	3.70	3.57	2.85
l Rol. operc. (OP 4, 50%)^3^	−60 0 7	–	3.87	3.12	2.22	3.50
l angular g./post. MTG	−48 −66 34	999	3.07 (2.61)	2.43	2.44	2.96
r planum temporale	42 −36 16	11475	4.60 (2.99)	3.74	4.40	3.90
r MTG	66 −24 −5	1053	3.44 (2.71)	3.81	3.19	2.85
r middle occipital gyrus	36 −84 31	4050	4.17 (2.88)	2.56	3.29	2.93
**Low ** ***E*** **_κ_>high ** ***E*** **_κ_**						
r V1 (area 17, 100%)	15 −99 −10	30915	4.24 (2.70)	3.54	3.19	3.13
r cerebellum^4^	27 −60 −44	–	4.23	3.76	4.15	3.13
WM	−18 9 34	14472	4.82 (2.73)	3.07	3.18	2.64
l LGB (Th-visual, 47%)^5^	−27 −27 −3	–	3.34	3.05	3.26	3.58
r (dorsal) striatum	23 14 9	14850	4.57 (2.80)	3.33	2.54	2.79
r LGB (Th-visual, 43%)^6^	27 −25 −3	–	3.87	3.36	3.41	4.15
WM	21 −24 46	1215	3.53 (2.79)	2.76	3.71	2.61

In the left column, percentages in brackets following anatomical structures refer to anatomical probabilities according to the SPM Anatomy Toolbox [102]. The next columns provide MNI-coordinates, cluster sizes, and *z*-values of maxima indicated by the analysis in which one single ECM per participant (calculated for the entire fMRI session) entered the second-level analysis. Results were corrected for multiple comparisons (*p*<.05). The three remaining columns on the right indicate maximal *z*-values in the respective structures when ECMs were computed separately for each emotion condition (joy, fear, and neutral, see Methods). Abbreviations: aFMC: anterior fronto-median cortex; g.: gyrus; l: left; parahipp.: parahippocampal; post.: posterior; r: right; ACC: anterior cingulate cortex; ECM: Eigenvector Centrality Mapping; IFG: inferior frontal gyrus; LGB: lateral geniculate body of the thalamus; MTG: middle temporal gyrus; Rol.operc.: Rolandic operculum; STG: superior temporal gyrus; SUB: subiculum of the hippocampal formation; Th-visual: visual thalamic nuclei; V1: primary visual cortex; WM: white matter.

1 The cluster with the peak voxel in the r parahipp. g. had another local maximum in the SUB.

2 The cluster with the peak voxel in the r ACC had another local maximum in the aFMC.

3 The cluster with the peak voxel in the l STG had another local maximum in the Rol. operc.

4 The cluster with the peak voxel in V1 had another local maximum in the cerebellum.

5 The cluster with the peak voxel in the WM had another local maximum in the l LGB.

6 The cluster with the peak voxel in the r striatum had another local maximum in the r LGB.

To investigate whether these differences were dependent on the emotional states of subjects, we also computed average ECMs for each emotion condition separately (see Methods). Comparisons between *E*_κ_ groups showed that, in almost all structures listed in Table 3, the significance values of the observed correlations were similar for joy, fear, and neutral (see Table 3 for details), except in the right subiculum (in which neutral stimuli did not evoke significant effects) and the left pars orbitalis (in which joy stimuli did not evoke significant effects).

### Discussion

#### Functional MR data and psychological (NEO) measures

The ECM data showed a correlation between agreeableness scores and centrality values in the DLPFC bilaterally. Separate clusters were observed bilaterally in both the posterior superior frontal sulcus (SFS, see left upper panel of Figure 2a), and the superior frontal gyrus (all clusters were located in area 8, [79]). The SFS clusters closely resemble results reported by Haas et al. [17], in which agreeableness correlated with activity in the (right) DLPFC (crown of the right medial frontal gyrus) during the processing of fearful faces. The authors of that study [17] presumed that, because agreeableness is associated with the tendency to avoid interpersonal conflict, highly agreeable individuals engaged neural mechanisms of affect regulation when facing conflict-related signals. This notion is also consistent with the recent observation that DLPFC is involved in impulse control [80]. To investigate whether ECM clusters observed in the present study in the DLPFC were driven by the fearful musical stimuli only, we computed ECMs for each experimental condition (joy, fear, neutral music), and correlated the ECMs with agreeableness scores. Local maxima at virtually identical coordinates in the DLPFC were observed for all three conditions (although with lower statistical significance), and these local maxima had comparable *z*–values for fear and joy conditions. This renders it unlikely that only threatening stimuli led to the activation of computational hubs in the DLPFC correlating with agreeableness. Perhaps agreeableness is associated with executive functions in DLPFC due to the organization of behaviour in regard to cooperation and social harmony in any given situation with social relevance, but this remains to be specified.

Centrality values also correlated positively with agreeableness scores in the ventral striatum/nucleus accumbens (NAc). This is perhaps related to agreeableness representing a tendency toward pro-social behaviour (including altruism and cooperation). Note that the musical stimulus used in the functional experiment probably evoked a number of music-related social functions such as social cognition, communication, and premotor coordination of movements [81], [82]. Engagement in such functions has been proposed to lead to feelings of reward [83], involving dopaminergic activity in the ventral striatum [84], and such music-evoked social functions are possibly activated more strongly in individuals with higher agreeableness scores, resulting in increased reward-related brain activity.

No correlations between extraversion and ECMs were observed, perhaps because potential results have been missed due to the relatively small sample size of *n* = 22 [85]. Also note that, due to the sample size, the significant effects we observed might not be as selective as it appears [85].

#### Functional MR data and physiological (cardiac) measures

The group with higher *E*_κ_ values (taken to reflect higher tender emotionality) showed higher centrality values in the subiculum of the right hippocampal formation (the implications of this finding are discussed in the General Discussion). This observation is consistent with results of two previous fMRI experiments, in which fMRI data also showed differences within the hippocampal formation between individuals with high and low *E*_κ_ [46]. In that study [46], which used the traditional general linear model approach (GLM), the main group difference was located in the cornu ammonis. Note that the present study used ECM, thus calculating computational hubs that have high centrality in networks consisting of different brain structures. In contrast to the cornu ammonis, which primarily receives projections from the dentate gyrus and projects to the subiculum [86], the subiculum is interconnected with a large array of cortical and subcortical structures: In addition to converging input from the cornu ammonis, it has bidirectional projections with entorhinal, perirhinal and prefrontal cortices, as well as with many subcortical structures (in particular numerous hypothalamic nuclei) [87]. Thus, it is likely that GLM contrasts are better suited to reveal functional differences in the cornu ammonis, whereas ECM is better suited to reveal such differences in the subiculum.

The group with higher *E*_κ_ also showed higher centrality values in the ACC. Due to the association of *E*_κ_ with NEO-scores of extraversion, this finding is consistent with studies showing correlations with extraversion and ACC activity [12]–[14]. The anterior and middle cingulate cortex is involved in emotional processes, in particular with regard to autonomic regulation and the production of subjective feelings [88], [89]. In addition, the cingulate cortex has been implicated in the coordination, and synchronization of autonomic activity, behaviour, motor expression, as well as cognitive processes in response to emotionally salient stimuli [89], [90]. In the *component process model* of emotion by Scherer [91], an emotion is defined as synchronization of these emotion components, and the observation that centrality values in the ACC are higher in individuals with high *E*_κ_ is thus likely to reflect stronger, or more frequent, synchronization processes in the ACC due to more dynamic emotion-related activity in the “biological subsystems of emotion” [91]. Interestingly, the cluster observed in the ACC also extended into the anterior fronto-median cortex (in which individuals with higher *E*_κ_ values also showed higher centrality values). This possibly reflects that individuals with higher tender emotionality engage more strongly in social information processing, including understanding and evaluating emotions of other individuals.

Another interesting result is that the group with lower *E*_κ_ values showed higher centrality values in the lateral geniculate body (LGB) bilaterally as well as in the primary visual cortex (V1). Note that both groups had their eyes closed during stimulation (this was controlled via an eyetracking device, see *Methods*), and that activation of V1 and LGB with closed eyes is consistent with activation patterns during visual imagery [92]. Although not included in our hypotheses, this pattern thus indicates increased visual imagery in individuals with low *E*_κ_ (compared to individuals with high *E*_κ_). As mentioned in the Introduction, reduced tender emotionality, i.e. emotional coldness and reduced affect, is taken to be reflected in low *E*_κ_. In a clinically relevant form, emotional coldness and reduced affect are the prime symptoms of schizoid personality disorder according to the International Classification of Diseases (F60.1 of the ICD-10), which lists as further characteristics “preoccupation with fantasy and introspection”. Because fantasy involves visual imagery, our results support the notion that, in healthy individuals, low *E*_κ_ may reflect subclinical symptoms of schizoid personality disorder, including reduced tender emotion (i.e., increased emotional coldness) and increased tendency for fantasy and introversion. Notably, this is also reflected in the lower extraversion scores of individuals with low *E*_κ_.

#### Emotion-specificity of ECM results

In addition to ECMs computed for the entire fMRI session, we also computed ECMs for each stimulus condition separately (joy, fear, neutral) to investigate to which degree personality-characteristic brain activity might have been dependent on the emotional states of subjects. Results showed that effects observed in the analysis of the entire fMRI session (pooling the data of all music stimuli and emotion ratings) were also observed in each of the stimulus conditions (joy, fear, neutral). This indicates that the reported ECM findings were largely independent of the emotional states of subjects and that the ECM results on emotional personality are not specific to any particular emotion. It is also unlikely that the observed associations between personality and brain activity are limited only to emotional states involving musical stimulation. Future studies might investigate whether the differences observed in Experiment 1 can also be observed in emotionally neutral experimental paradigms.

## Experiment 2: Structural MRI Using Voxel Based Morphometry (VBM)

Experiment 1 investigated personality-characteristic differences in brain function. In Experiment 2, we aimed at investigating whether such differences correspond to differences in brain structure. For this purpose, the same psychological (NEO) and physiological (cardiac) measures were acquired from an independent sample of subjects and used as independent variables to explain variance in structural neuroimaging data. In addition, we hypothesized (based on the most consistent findings reported in previous studies, see Table 1B) positive correlations between neuroticism and OFC volume [26], [27] as well as between neuroticism and amygdalar volume [23], [28], [29]. Likewise, we aimed to test for positive correlations between extraversion and OFC volume [20]–[22] as well as amygdalar volume [21], [23], and to test for a negative relation between extraversion and volume of the caudate nucleus [25], [26].

### Materials and methods

#### Ethics statement

All subjects gave written informed consent. The study was conducted according to the Declaration of Helsinki and approved by the ethics committee of the School of Life Sciences and the Psychology Department of the University of Sussex.

#### Participants

Data were acquired from 59 individuals (aged 20–31 years, *M* = 24.15, *SD* = 2.40, 34 females). None of the subjects participated in Experiment 1. Exclusion criteria were left-handedness, past diagnosis of a neurological or psychiatric disorder, and abnormal brain anatomy.

#### Experimental procedure

Acquisition and analysis of psychological (NEO) measures and physiological (cardiac) measures was identical to Experiment 1, except that ECG and NEO data were obtained within three weeks before or after the structural imaging session, and that ECG data were acquired using a Refa-system (Twente Medical Systems, Enschede, NL).

#### MR scanning

Scanning was performed with a 3T Siemens Magnetom Trio scanner (Siemens Medical Systems, Erlangen, Germany). Whole-brain structural scans were acquired using a 3D Fast Low Angle Shot (FLASH) sequence using a 1-channel birdcage head coil. For each subject, 176 sagittal partitions with an image matrix of 240×256 and an isotropic spatial resolution of 1 mm, a TE = 4 ms, α = 10° and total scan time of about 8 min were acquired.

#### Structural MRI data analysis

Processing of structural MRI scans was carried out using SPM8 [93] running on Matlab 2009a (The Mathworks, Natick, MA, USA). All images were checked for artefacts and manually aligned so that the origin of the coordinate system was located at the anterior commissure. Using the *New Segmentation* procedure of SPM8 [94], which is an extension of the default unified segmentation procedure [95], the images were segmented into grey matter (GM), white matter (WM) and cerebrospinal fluid (CSF). For each subject, this resulted in two sets of 3 images: One set was in the same space as the original T1-weighted image in which each voxel was assigned a probability of it being GM, WM and CSF; another set contained the segmented images rigid-body-aligned into MNI space. Spatial normalization employed DARTEL, an algorithm for diffeomorphic image registration [96] that provides an improved anatomical precision [97]–[99] compared to previously used methods [95], [100]. DARTEL was used to optimally warp the rigid-body aligned GM, and WM segments into a new reference space representing an average of all the participants. The method iteratively creates an increasingly fine-grained set of group-specific templates and the deformation fields required to warp the data from each subject. Each subject's specific deformation field is used to warp the corresponding GM and WM segments into the new reference space, with re-sampling at 1.5 mm isotropic voxels using trilinear interpolation. Then, the warped GM and WM segments were affine transformed into MNI space. The probability values were scaled by the Jacobian determinants of the deformations to account for the local compression and stretching that occurs as a consequence of the warping and affine transformation (a process referred to as *modulation*) [101]. Finally, the GM probability values were smoothed using a 4 mm FWHM Gaussian kernel.

The GM volumetric data were then correlated voxel-wise with each of the (standardized) NEO scores (extraversion, neuroticism, agreeableness) with an FWE-correction for multiple comparisons (*p*<.05; NEO-scores did not deviate from a standard normal distribution as indicated by a Shapiro-Wilk-Test, see Table S2). As in the functional experiment, *E*_κ_ values deviated from a normal distribution (*p* = 0.001 according to the Shapiro-Wilk Test of Normality). Therefore, *E*_κ_ values were dichotomized using a median-split, and GM volumetric data were compared between the resulting two groups (33 individuals with higher, and 34 individuals with lower *E*_κ_) using voxel-wise *t*–tests and an FWE-correction for multiple comparisons (*p*<.05). In cases where we had directed hypotheses (see Introduction of Experiment 2), we also computed uncorrected SPMs (thresholded at *p* = .001 and a voxel extent of 10 voxels). In all analyses, we controlled for differences in age, gender and total brain volume (sum of GM and WM volume) by including these variables as covariates of no interest.

### Results

#### Relations between ECG and NEO data

As in the functional experiment, NEO scores were compared between groups with high and low *E*_κ_ values (i.e., *E*_κ_ values above and *E*_κ_ values below the median) according to the directed hypotheses stated in the Introduction. There was a trend for individuals with higher *E*_κ_ showing higher extraversion scores compared to those with lower *E*_κ_ (*t* = 1.7, *p* = .047, one-tailed). Contrary to the functional experiment, individuals with higher *E*_κ_ did not show significantly higher scores of positive emotion (for details and complete data see Table S3).

#### Psychological (NEO) measures and VBM data

**Figure 3:**
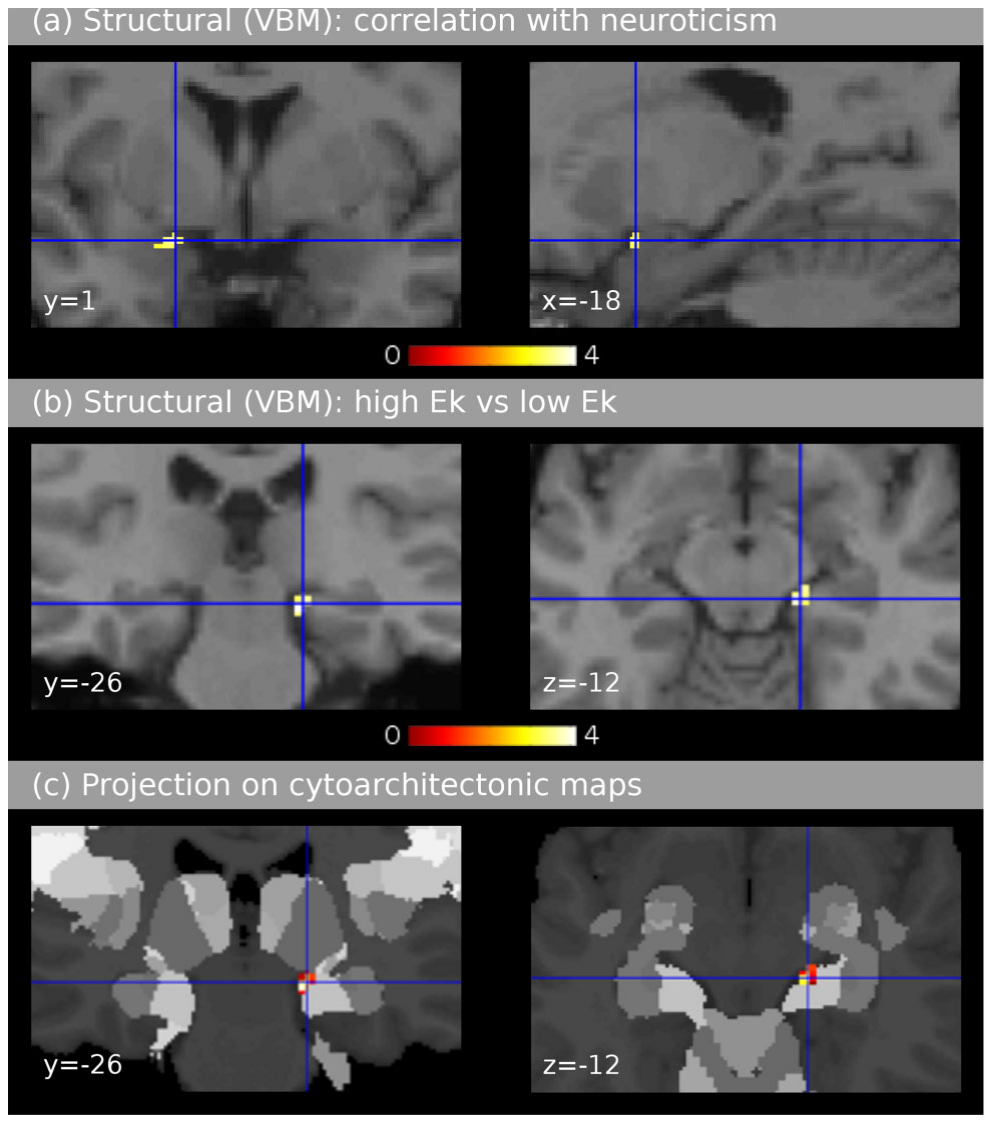
Experiment 2: Structural neuroimaging data (voxel-based morphometry). Results were controlled for total brain volume, age and gender, images are shown in neurological convention. The upper panel (**a**) shows results of the correlations of grey matter volume with neuroticism scores (uncorrected data with a threshold of *p*<.001 and a minimum cluster size of 10 voxels). The crosshair indicates a positive correlation in the left amygdaloid complex (significant in the region of interest analysis, *p*<.05, FWE-corrected). The left image shows a coronal section, the right image shows a sagittal section. The middle panel (**b**) shows the comparison of structural data between groups of participants with higher and lower *E*_κ_ values (uncorrected data with a threshold of *p*<.001 and a minimum cluster size of 10 voxels). A difference between groups was found in the subiculum of the right hippocampal formation (significant in the region of interest analysis, *p*<.05, FWE-corrected). Gray matter volume was larger in the group of participants with higher *E*_κ_ values (taken to reflect higher tender emotionality). Note the consistency with the group difference observed in the left subiculum in the functional data (Figure **2**b). The left image shows a coronal section, the right image an axial section. The bottom panel (**c**) shows the same results as (**b**), projected on the cytoarchitectonic probability map of the hippocampal formation provided by Amunts et al. [78] using the Anatomy Toolbox provided by Eickhoff et al. [102]. The group difference is located with 90% probability in the subiculum.

In a whole-brain-analysis, voxel-wise correlations between the structural brain data (controlled for total brain volume, age and gender) and each of the NEO scales did not indicate any significant correlation in the FWE-corrected statistical parametric maps (SPMs). Inspecting the uncorrected SPMs (based on our directed hypotheses) we observed a positive correlation between neuroticism and tissue density in the left amygdala (Figure 3a). When applying a small volume correction, using the amygdala volume as region of interest (extracted from the Anatomy Toolbox for SPM) [102] this correlation was significant at the FWE-corrected threshold (*p*<.05 on both cluster and peak level). The voxel with the maximum *z*–value (3.61) was located at MNI coordinate −18, 1, −17.

#### Physiological (cardiac) measures and VBM data

In another whole-brain analysis, we computed a voxel-wise comparison of structural brain data (controlled for total brain volume, age and gender) between the two *E*_κ_ groups (*E*_κ_ values above and *E*_κ_ values below the median). The FWE-corrected SPM of this comparison did not indicate any significant group difference. Again, inspecting the uncorrected SPMs (driven by our directed hypothesis) to investigate possible group differences in amygdala and hippocampal formation, we observed that the largest cluster in the comparison high>low *E*_κ_ was located in the subiculum of the right hippocampal formation (Figure **3**b), in part overlapping with the cluster that was observed to differ between groups in the functional data. When using the volume of the subiculum as region of interest (subiculum volume was extracted from the Anatomy Toolbox for SPM) [102], this volume difference between groups was significant after FWE-correction (*p*<.05 on the cluster level, and *p*<.006 on the peak level). When using the entire hippocampal volume (bilaterally) as region of interest, the difference between groups was significant on the peak level (*p*<.05, FWE-corrected). The voxel with the maximum *z*–value (3.75) was located at MNI-coordinate 18, −26, −12 (90% probability for subiculum of the hippocampal formation according to the cytoarchitectonic maps provided by Amunts et al., [78], see also Figure 3c). The opposite uncorrected contrast (low>high *E*_κ_ values) did not yield any significant volume differences between groups.

### Discussion

#### Structural MR data and psychological (NEO) measures

The positive correlation between neuroticism and volume of the left amygdala corroborates findings of a previous study showing a correlation between neuroticism and amygdalar volume (in females) [28]. Another previous finding of an association between neuroticism and right amygdalar volume [23] was not supported by our study. It is well established that the amygdala is involved in fear responses [103], and the correlation between neuroticism and amygdalar volume might thus reflect structural changes in the amygdala due to the increased tendency to experience anxiety. Corroboratingly, the amygdaloid complex (and related orbitofrontal cortical areas) is centrally involved in mood disorders such as major depressive disorder, bipolar disorder, and pathological anxiety [104], [105]. However, substantial evidence indicates that the amygdala also plays a role for positive emotions [106]. Therefore, while amygdalar nuclei involved in negative emotion might have greater volume in individuals with high neuroticism, one could also expect greater volume in nuclei involved in positive emotion in individuals with low neuroticism, or high agreeableness. Perhaps negative emotions lead to more pronounced structural changes in the amygdala than positive emotions, but this remains to be specified. No correspondence between functional (Experiment 1) and structural MRI results (Experiment 2) was observed with regard to the analyses involving NEO measures, substantiating that consistency of neuroimaging results using self-report (questionnaire) measures or personality as independent variables is rather low [107] (see also Table 1).

#### Structural MR data and physiological (cardiac) measures

The group with higher *E*_κ_ (compared to the group with lower *E*_κ_) showed larger volume in the subiculum of the right hippocampal formation. These are the first results showing a relation between a cardiac amplitude signature and brain morphometry. Both structural and functional results correspond remarkably well with each other: Effects were partly overlapping, and the maximal effects were located in close vicinity across Experiments 1 and 2. This provides strong evidence for an association between the hippocampal formation and cardiac amplitude signatures in humans. Because the associations between NEO scores and *E*_κ_ corroborate a relation between *E*_κ_ and emotional personality, these structural data are also the first to show that emotionality-related differences as assessed with a peripheral physiological measure correlate with brain morphology.

## General Discussion

### Summary of results

The group with higher *E*_κ_ values (taken to reflect higher tender emotionality) had higher extraversion scores in both the functional and the structural experiment. In the functional experiment, scores of positive emotion were higher in the group with higher *E*_κ_ values. The functional data indicated a significant positive correlation between centrality values and agreeableness in the right ventral striatum (probably nucleus accumbens, NAc), ACC, the rostral cingulate zone extending into the pre-SMA, and dorsolateral prefrontal cortex (DLPFC) bilaterally. Significant negative correlations were found in the central sulcus. The comparison of the functional data between the two *E*_κ_ groups showed that, within the subiculum of the right hippocampal formation, centrality values were higher for the group with higher *E*_κ_ values. Moreover, significantly higher centrality values in the group with higher *E*_κ_ values were indicated in the auditory cortex bilaterally, in both anterior and posterior cingulate cortex, the anterior fronto-median cortex, and the Rolandic operculum. The group with lower *E*_κ_ values showed higher centrality values in the lateral geniculate body bilaterally as well as in the visual cortex (V1). The analysis of the structural brain data showed a positive correlation between neuroticism and volume of the left amygdala in a ROI analysis of the amygdala. A comparison of the structural data between individuals with high and low *E*_κ_ values revealed a group difference in the right subiculum of the hippocampal formation in a ROI analysis (individuals with high *E*_κ_ values had larger grey matter volume).

### Relations between cardiac and NEO measures

In each experiment the group with higher *E*_κ_ values had higher extraversion scores. This replicates results of a previous experiment [47], thus up to now data from three independent samples show higher extraversion scores in individuals with higher *E*_κ_ values. In addition, as in our previous study, scores of positive emotion (a facet of extraversion) were higher in the group with higher *E*_κ_ values in Experiment 1 (although not in Experiment 2). These findings support the notion that *E*_κ_ reflects aspects of the emotional personality of an individual. This notion is also corroborated by the behavioral ratings about the emotional states of participants (obtained after each stimulus in Experiment 1). These emotion ratings showed that *E*_κ_ was related to felt arousal and fear. However, as a note of caution, the relation between *E*_κ_ and neuroticism observed in our previous study [47] was not replicated by the present results, and contrary to our hypotheses, our results did not show relations between *E*_κ_ values and tender-mindedness, nor between *E*_κ_ values and warmth (although these two NEO facets are conceptually related to tender emotionality). Hence, *E*_κ_ does not reflect “friendliness towards others”, and “sympathy for others” as measured with the NEO-PI-R. Moreover, the present results call the relation between *E*_κ_ and neuroticism (as previously observed [47]) into question. To further investigate to which degree *E*_κ_ reflects tender emotionality, future studies might thus better use questionnaires that are more specifically designed to assess positive emotion, attachment-related emotions, compassion and empathy.

As outlined in Box 1, personality-specific modulation of regional cardiac activity is likely due to the influence of psychological factors on activity of neurons within the cardiac nerve plexus. Such influence includes (a) autonomic activity and sympathovagal balance (a major reaction component of emotion, and thus of emotional personality), (b) limbic/paralimbic modulation of efferent autonomic outflow to the heart, (c) hemispheric weighting of emotional activity and corresponding asymmetric autonomic outflow, as well as (d) central nervous system processes that regulate circulating hormones [108]. The exact mechanisms involved in psychological modulation of regional heart activity are yet unknown.

Notably, compared to personality questionnaires, the cardiac measure used in the present study does not face the problem of potential subjective bias such as socially desirable responding, inaccuracies in self-perception, self-favouring tendencies, self-deception, and moralistic bias [33]–[37] (although, on the other hand, this cardiac measure may well be influenced by biological factors unrelated to personality, as mentioned in the Introduction). Therefore, combinations of biomarkers such as the cardiac measure used in the present study, or similar cardiac indices [47], with standardized personality questionnaires might lead to a substantial improvement in the assessment of personality and in the investigation of neural correlates of emotional personality. To investigate a possible gain from combining questionnaire and ECG measures of personality, future studies could obtain data from healthy controls and from patients (e.g., from individuals with depression, or with a personality disorder such as schizoid personality disorder) and investigate whether the combination of NEO and ECG scores (as compared to using either only ECG or only NEO scores) significantly increases the discrimination of the two groups.

### The functional significance of the hippocampal formation for emotional personality

The present results reveal a link between regional cardiac activity on the one hand, and both function as well as structure of the subiculum of the hippocampal formation on the other. Regional cardiac activity was reflected in the *E*_κ_ values, i.e. in the relations of ECG amplitudes recorded at different ECG leads (cf. Figure 1). It is unlikely that the relations of amplitudes as captured in *E*_κ_ are simply due to different levels of cardiovascular stress: body mass index (BMI) was in both groups within the normal range (mean BMI, averaged across all participants of Experiments 1 and 2, was 21.8 [SD = 2.9] in the group with higher, and 23.0 [SD = 3.0] in the group with lower *E*_κ_ values, with no significant difference between groups), and both groups consisted of young adults without cardiovascular disease. This also renders it likely that blood pressure did not differ between groups (similar to our previous study [46]), although blood pressure was not obtained in the present study. In addition, our previous investigations [46] indicated that *E*_κ_ values do not correlate with serum concentrations of N-terminal pro-brain natriuretic peptide (and are thus not related to left-ventricular hypertrophy). Hence, the association between *E*_κ_ values and hippocampal activity does not appear to originate simply from different levels of cardiovascular stress. Instead, in light of the associations between *E*_κ_ values and personality measures observed in the present as well as in previous studies, the present data corroborate the notion that the hippocampal formation plays a role for emotional personality.

In cognitive neuroscience, the hippocampus is best known for its role for learning and memory [109], as well as for novelty and expectedness [110]. In addition, however, a plethora of evidence indicates that the hippocampus also plays an important role for emotional processes: For example, the hippocampus is substantially involved in the regulation of the hypothalamic-pituitary-adrenal (HPA) axis stress response [111], and hippocampal neurons are uniquely vulnerable to emotional stressors [112]. Particularly the subiculum is substantially involved in the regulation of HPA axis activity, and profoundly affected by emotional stress [87]. The fact that the subiculum has dense connections to numerous hypothalamic nuclei (in addition to connections with many other subcortical and cortical structures) [87] makes it highly likely that the subiculum has modulatory influence on cardiac activity via endocrine and autonomic neurons located in the hypothalamus. Therefore, physiology lends plausibility to the notion that, in humans, emotional personality is at least in part associated with hippocampal activity, and that such activity modulates regional heart activity. The notion that the hippocampal formation is a neural substrate involved in tender emotion is consistent with the view that the hippocampal formation generates attachment-related affects that are perceived by humans as tender feelings [81]. Attachment-related behaviour includes kissing, caressing, hugging, softly touching, softly vocalizing, and in animals behaviours such as licking, grooming, nest-building, and pup retrieval. Several lines of evidence point to the involvement of the hippocampus in attachment-related emotions: (1) Lesions of the hippocampus lead to impairment of maternal behaviour in rats as indexed by less frequent and less efficient nursing, poorer nest building, increased maternal cannibalism, poorer pup retrieving, and fewer pups surviving to weaning [113]. On the other hand, increased pup licking, grooming and nursing by rat mothers leads to increased hippocampal glucocorticoid receptor messenger RNA expression in the offspring [114], influences hippocampal synaptic development in the offspring [115], and alters the offspring epigenome at a glucocorticoid receptor gene promoter in the hippocampus [116]. (2) The hippocampus is damaged by chronic emotional stressors, particularly by helplessness and despair [112]. The hippocampus is unique in its vulnerability to emotional stressors, and presumably the only brain structure in which severe emotional stress can lead to the loss of neurons (in addition to neuronal death, the volume reduction of the hippocampus in response to severe chronic emotional stress is due to reduced neurogenesis in the dentate gyrus of the hippocampal formation). (3) Dysfunction and structural damage of this structure has been observed in individuals suffering from post-traumatic stress disorder (PTSD) such as Vietnam veterans who witnessed extreme violence or committed extremely violent acts [117]; similar findings have been reported for individuals that were sexually abused during childhood [118]. Moreover, both dysfunction and volume reduction of the hippocampal formation has been observed in depressive patients [119]. Our results show that even in healthy, non-clinical individuals, hippocampal volume and hippocampal activity is related to regional heart activity as reflected in *E*_κ_ values (and, thus, presumably to tender emotionality). Whether the group differences in hippocampal volume are due to (subclinical) trauma in individuals with low *E*_κ_, or due to use-dependent hippocampal plasticity in individuals with higher *E*_κ_, remains to be specified. (4) Numerous functional neuroimaging studies with healthy adults indicate signal changes in the hippocampal formation in response to pleasurable, joyful music [80]. This association between hippocampal activity and music-evoked feelings of joy has been linked in part to the social functions of music, such as communication, cooperation, and social cohesion, thus to functions that serve the formation of interindividual attachments.

Group differences in the hippocampal formation were observed in the right hemisphere, in both functional and structural experiments of the present study, as well as in two previous functional neuroimaging experiments [46]. As mentioned in the Introduction, the left and right hemispheres of the human forebrain have different associations with particular emotions and affective traits [57], probably due to an asymmetrical representation of homeostatic activity that originates from asymmetries in the peripheral autonomic nervous system [52], [58], [59]. Lesion studies indicate a right-hemispheric dominance for sympathetic efferent neuronal effects, and stimulation of the right anterior insular cortex elicits increases in heart rate and blood pressure (whereas left anterior insular stimulation decreases heart rate) [52], [58], [59]. Thus, the right-lateralization of hippocampal activity possibly originates from a more dynamic emotional activity involving increased sympathetic tone in individuals with high *E*_κ_. This notion is supported by two previous experiments showing higher heart-rate variability in individuals with high *E*_κ_ (compared to individuals with low *E*_κ_) [46], [47], indicating a stronger tone of the sympathetic branch of the autonomic nervous system in individuals with high *E*_κ_.

### Limitations

Although the present results corroborate relations between *E*_κ_ and psychological (NEO) measures of personality (in both experiments, individuals with higher *E*_κ_ had higher extraversion scores, as in previous studies), it remains arguable to which degree *E*_κ_ is a valid measure of tender emotionality. This could, for example, be investigated further in studies that perform diagnostic interviews, conducted by independent raters, to test whether a classification based on *E*_κ_ corresponds to the interview-based classification of tender emotionality.

Moreover, it has previously been noted [85] that in experiments with relatively small sample sizes (such as n = 22, as in our functional experiment), only a fraction of the effects that really exist might be detected, and that results might promote the illusion of highly selective activations. Thus, as noted above, the effects we observed in the functional experiment might not be as selective as observed, and there might be more potential results that have been missed due to the relatively small sample size.

### Conclusions

This study has three main conclusions: *First*, our data show personality-characteristic computational hubs in the brain (identified using Eigenvector Centrality Mapping). Results substantiate previous studies reporting associations between agreeableness and function of the DLPFC, as well as between tender (positive) emotionality and (right) hippocampal function. In addition, they reveal an association between agreeableness and function of the ventral striatum (including the nucleus accumbens), possibly related to the rewarding nature of altruism and cooperation. *Second*, the structural data substantiate associations between neuroticism and amygdalar volume. Moreover, the structural data show an association between volume of the hippocampal formation and regional cardiac activity taken to reflect emotional personality. The latter association was revealed in virtually the same aspect of the right subiculum as in the functional data. Thus, along with previous reports on this issue, there is substantial convergence and replicability of functional and structural associations between a cardiac measure related to emotional personality and the hippocampal formation. This indicates a new role of the hippocampal formation in human emotional personality (in addition to its well-known role for cognition). Previous studies showed associations between structure and function of the hippocampal formation in clinical populations (such as patients with depression, PTSD, or borderline personality disorder). Our results indicate that in healthy individuals function and structure of the hippocampal formation is associated with human emotional personality. *Third*, results demonstrate that based on a cardiac measure, consistent and replicable results can be obtained in the analysis of functional as well as structural neuroimaging data, in addition to replicable associations between this cardiac measure and extraversion (and, in part, positive emotion) as obtained with the NEO inventory. This substantiates that ECG amplitude relations are useful biomarkers for effects of emotional personality on regional heart activity. The replicability and consistency of results obtained with the cardiac index used in the present study (as compared with results obtained using personality questionnaires) motivates further investigation of the cardiac indices of personality, and the combination of psychological and physiological measures in the assessment of emotional personality.

## Supporting Information

Methods S1 **Experiment 1: Description of stimuli.**
(DOC)

Table S1 **List of stimuli.**
(DOC)

Table S2 **Descriptive statistics of questionnaire scores.**
(DOC)

Table S3 **Statistics of NEO scales (neuroticism, extraversion, agreeableness) and NEO facets (warmth, positive emotion, tender-mindedness).**
(DOC)
