# Supplementary material for: Neural Correlates of Emotional Personality: A Structural and Functional Magnetic Resonance Imaging Study
Source: PLoS One. 2013 Nov 27;8(11):e77196. doi: 10.1371/journal.pone.0077196 (PMC3842312; doi:10.1371/journal.pone.0077196)
Supplement: Methods S1 — Experiment 1: Description of stimuli. (DOC) [file pone.0077196.s001.doc]

## Supporting Methods

### Experiment 1: Description of stimuli

Musical stimuli were selected to evoke (a) feelings of joy, (b) feelings of fear, or (c) neither joy nor fear (henceforth referred to as neutral stimuli). There were *n*=8 stimuli per category, and each stimulus was presented twice during the experiment. The complete list of joyful and fearful stimuli is provided in Supplementary Table S1. Behavioral data showed that musical stimuli evoked the intended feelings in our study population: Mean joy ratings (with standard deviation in parentheses), on a scale from 1 = evokes no joy at all to 6 = evokes very strong joy, were 4.90 (0.43) for joy stimuli, 2.45 (0.29) for neutral stimuli, and 1.68 (0.16) for fear stimuli. Mean fear ratings (with standard deviation in parentheses), on a scale from 1 = evokes no fear at all to 6 = evokes very strong fear, were 3.92 (0.21) for fear stimuli, 2.26 (0.32) for neutral stimuli, and 1.26 (0.16) for joy stimuli.

Joyful stimuli had been used in previous studies [1-4] and consisted of CD-recorded pieces from various epochs and styles (classical music, Irish jigs, jazz, reggae, South American and Balkan music). Fearful musical stimuli were excerpts from soundtracks of suspense movies and video games. To increase the fear-evoking effect of the fear stimuli, their relatively high acoustic roughness was further increased by creating dissonant versions, where each musical excerpt was audible in three different pitches simultaneously (original pitch, one semitone higher, and a tritone lower) [3-5]. The pitch-shifted, additional counterparts of each excerpt were created so that only their pitch differed, while their tempo remained equal. The original excerpt together with the two pitch-shifted counterparts was then rendered as a single wav-file using Ableton Live (version 8.0.4, Ableton Inc., New York, USA). Neutral stimuli were sequences of isochronous tones, for which the pitch classes were randomly selected from a pentatonic scale. Tone sequences were coded in MIDI (musical instrument digital interface) and generated using the MIDI toolbox for Matlab [Error: Reference source not found]. Importantly, for each joyful-fearful stimulus pair, a neutral control stimulus was generated that matched joyful and fearful stimuli with regard to tempo, F0 (i.e. fundamental frequency) pitch range, and instrumentation (using the two respective main instruments or instrument groups of the respective joyful-fearful pair). To create stimuli that sounded like musical compositions played with real instruments (similar to the joyful and fearful stimuli), the tones from the MIDI sequences were set to trigger instrument samples from a high quality natural instrument library (X-Sample Chamber Ensemble, Winkler & Stahl GbR, Detmold, Germany) and from the Ableton Instrument library (Ableton Inc., New York, USA). Stimuli were then rendered as wav-files using Ableton Live. Using Praat (version 5.0.29; Paul Boersma & David Weenink, 2009, Praat: doing phonetics by computer, http://www.praat.org), all excerpts (joyful, fearful, and neutral) were edited so that they all had the same length (30 s), 1.5 s fade-in/fade-out ramps, and the same RMS power.

Importantly, joyful and fearful stimuli were chosen such that each joyful excerpt had a fearful counterpart that matched with regard to tempo (beats per minute), mean F0 pitch, F0 pitch variation, pitch centroid value, spectral complexity, and spectral flux. This was confirmed by an acoustic analysis of the stimuli using *Essentia*, a library for extracting acoustical and music features from audio files (http://mtg.upf.edu/technologies/essentia). The Essentia software was also used to test for differences between stimuli with regard to other acoustical factors: 177 acoustical descriptors were extracted frame-by-frame (frame length = 21.5 ms, 50% overlap), averaged along the entire duration of the file, and then compared between conditions (joyful, neutral, fearful) using one-way ANOVAs. Significant effects of condition (Bonferroni-corrected significance-level was *p*<.001) were indicated for the following acoustic factors (with *F*−values in parentheses, degrees of freedom: 2, 21): (a) Mean (72.3) and variance (13.8) of F0 *salience* (this measure is highest for single tones, intermediate for chords, and lowest for noises; note that mean F0 and variance of F0 did not differ between joyful, fearful, and neutral stimuli). The mean F0 salience was highest for neutral, intermediate for joyful, and lowest for fearful stimuli (*p*<.001 in all pairwise comparisons). This reflects that both joyful and fearful (but not neutral) stimuli contained numerous harmonies, and that fearful (but not joyful) stimuli contained numerous percussive sounds, as well as hissing and whooshing noises. (b) Mean (41.3) and variance (28.0) of sensory dissonance. Sensory dissonance was lowest for neutral, intermediate for joyful, and highest for fearful stimuli. Mean sensory dissonance differed significantly between joyful and neutral (*p*<.001), between fearful and neutral (*p*<.001), and between joyful and fearful stimuli (*p*<.05). (c) Mean chord strength (25.2) and key strength (14.7); these factors measure how strongly a sound resembles the sound of a chord, and how clearly the sounds of a stimulus can be attributed to a key. Chord strength was larger for joyful compared to fearful, as well as for joyful compared to neutral sounds (*p*<.001 in each test), whereas fearful and neutral stimuli did not differ significantly from each other. Key strength was larger for joyful compared to fearful (*p*<.001), and for neutral compared to fearful sounds (*p*=.01); joyful and neutral stimuli did not differ significantly from each other (*p*>.15). (d) Mean (30.0) and variance (16.4) of spectral flux, mean spectral crest (30.0) and mean spectral complexity (10.6). Mean spectral flux, spectral crest, and spectral complexity were lowest for neutral stimuli (with significant differences between neutral and joyful, as well as between neutral and fearful stimuli, *p*<.05 in each test), and did not differ significantly between joyful and fearful stimuli (*p*>.2 in each test).

[1] Koelsch S, Offermanns K, Franzke P (2010) Music in the Treatment of Affective Disorders: An Exploratory Investigation of a New Method for Music-Therapeutic Research. Music Perception 27: 307–316.

[2] Fritz T, Jentschke S, Gosselin N, Sammler D, Peretz I, et al. (2009) Universal recognition of three basic emotions in music. Current Biology 19: 573–576.

[3] Koelsch S, Fuermetz J, Sack U, Bauer K, Hohenadel M, et al. (2011) Effects of music listening on cortisol levels and propofol consumption during spinal anesthesia. Frontiers in Psychology 2: 1-9.

[4] Koelsch S, Skouras S, Fritz T, Herrera P, Bonhage C, Küssner MB, Jacobs AM (2013) Neural correlates of music-evoked fear and joy: The roles of auditory cortex and superficial amygdala. NeuroImage 81: 40-60.

[5] Koelsch S, Fritz T, Cramon DY, Müller K, Friederici AD (2006) Investigating emotion with music: An fMRI study. Human Brain Mapping 27: 239–250.
