# Supplementary material for: Neural Correlates of Emotional Personality: A Structural and Functional Magnetic Resonance Imaging Study
Source: PLoS One. 2013 Nov 27;8(11):e77196. doi: 10.1371/journal.pone.0077196 (PMC3842312; doi:10.1371/journal.pone.0077196)
Supplement: Table S2 — Descriptive statistics of questionnaire scores. (DOC) [file pone.0077196.s003.doc]

**Supporting Table S2. Descriptive statistics of questionnaire scores.**

|  | M | SEM | PR | p |
| --- | --- | --- | --- | --- |
| *Experiment 1 (fMRI)* |  |  |  |  |
| Neuroticism | 102.27 | 3.23 | 11.0 – 096.5 | 0.104 |
| Extraversion | 107.05 | 3.13 | 5.3 – 098.7 | 0.898 |
| Agreeableness | 112.64 | 3.29 | 13.9 – 100.0 | 0.506 |
| *Experiment 2 (sMRI)* |  |  |  |  |
| Neuroticism | 102.43 | 1.89 | 4.9 – 099.6 | 0.231 |
| Extraversion | 106.01 | 2.24 | 0.0 – 100.0 | 0.308 |
| Agreeableness | 103.90 | 1.68 | 5.3 – 098.7 | 0.370 |

Abbreviations: M: arithmetic mean; SEM: standard error of mean; PR: percentile rank of the scores; p: probability of deviation from normality tested using Shapiro-Wilk test.
