# Supplementary material for: Neural Correlates of Emotional Personality: A Structural and Functional Magnetic Resonance Imaging Study
Source: PLoS One. 2013 Nov 27;8(11):e77196. doi: 10.1371/journal.pone.0077196 (PMC3842312; doi:10.1371/journal.pone.0077196)
Supplement: Table S3 — Statistics of NEO scales (neuroticism, extraversion, agreeableness) and NEO facets (warmth, positive emotion, tender-mindedness). (DOC) [file pone.0077196.s004.doc]

**Supporting Table S3.** Statistics of NEO scales (neuroticism, extraversion, agreeableness) and NEO facets (warmth, positive emotion, tender-mindedness).

| **NEO-scale / facet** | **High *E*_κ_** | **Low *E*_κ_** | ***t*−(*p*−)values** |
| --- | --- | --- | --- |
|  |  |  |  |
| Neuroticism (Exp. 1) | 100.9 (3.7) | 100.6 (4.5) | 0.04 (*n*.*s*.) |
| Neuroticism (Exp. 2) | 98.1 (1.8) | 99.7 (2.0) | .63 (*n*.*s*.) |
| Extraversion (Exp. 1) | 111.8 (3.2) | 98.0 (4.5) | 2.50 (0.01) |
| Extraversion (Exp. 2) | 88.8 (1.3) | 85.6 (1.3) | 1.74 (0.05) |
| Agreeableness (Exp. 1) | 111.9 (3.8) | 108.8 (3.9) | .57 (*n*.*s*.) |
| Agreeableness (Exp. 2) | 100.6 (2.3) | 99.1 (1.5) | .51 (*n*.*s*.) |
| Warmth (Exp. 1) | 58.4 (3.6) | 52.4 (3.3) | 1.23 (*n*.*s*.) |
| Warmth (Exp. 2) | 33.5 (0.8) | 35.07 (0.8) | 1.40 (*n*.*s*.) |
| Positive Emotion (Exp. 1) | 55.3 (2.1) | 47.0 (2.6) | 2.54 (0.01) |
| Positive Emotion (Exp. 2) | 39.9 (0.6) | 40.8 (0.8) | .89 (*n*.*s*.) |
| Tender-mindedness (Exp. 1) | 53.9 (3.2) | 53.5 (2.9) | .11 (*n*.*s*.) |
| Tender-mindedness (Exp. 2) | 33.7 (0.9) | 35.9 (1.2) | 1.49 (*n*.*s*.) |

Data are shown separately for the groups with higher and lower *E*_κ_ values, and separately for the functional experiment (Exp. 1) and the structural experiment (Exp. 2). Scores of neuroticism, extraversion, and agreeableness are standard scores. Scores of warmth, positive emotion, and tender-mindedness are *T*-scores. The outermost right column shows *t*−values (with *p*−values in parentheses) for the comparison between groups with higher and lower *E*_κ_ values. Degrees of freedom for the group comparisons were 20 in Exp. 1, and 57 for Exp. 2.
